# Feasibility of a 3D-printed anthropomorphic patient-specific head phantom for patient-specific quality assurance of intensity-modulated radiotherapy

**DOI:** 10.1371/journal.pone.0181560

**Published:** 2017-07-20

**Authors:** Ji Woon Yea, Jae Won Park, Sung Kyu Kim, Dong Youn Kim, Jae Gu Kim, Chan Young Seo, Won Hyo Jeong, Man Youl Jeong, Se An Oh

**Affiliations:** 1 Department of Radiation Oncology, Yeungnam University Medical Center, Daegu, Korea; 2 Department of Radiation Oncology, Yeungnam University College of Medicine, Daegu, Korea; 3 Gyeongnam Science High School, Gyeongnam, Korea; Northwestern University Feinberg School of Medicine, UNITED STATES

## Abstract

This study evaluated the feasibility of utilizing a 3D-printed anthropomorphic patient-specific head phantom for patient-specific quality assurance (QA) in intensity-modulated radiotherapy (IMRT). Contoured left and right head phantoms were converted from DICOM to STL format. Fused deposition modeling (FDM) was used to construct an anthropomorphic patient-specific head phantom with a 3D printer. An established QA technique and the patient-specific head phantom were used to compare the calculated and measured doses. When the established technique was used to compare the calculated and measured doses, the gamma passing rate for γ ≤ 1 was 97.28%, while the gamma failure rate for γ > 1 was 2.72%. When the 3D-printed patient-specific head phantom was used, the gamma passing rate for γ ≤ 1 was 95.97%, and the gamma failure rate for γ > 1 was 4.03%. The 3D printed patient-specific head phantom was concluded to be highly feasible for patient-specific QA prior to complicated radiotherapy procedures such as IMRT.

## Introduction

The effectiveness of radiotherapy is maximized by delivering the highest possible dose of radiation to a tumor while minimizing the effect on normal tissues [1,2]. In particular, treatment techniques such as intensity-modulated radiotherapy (IMRT) and volumetric-modulated arc therapy (VMAT) optimize the composite dose distribution and deliver a non-uniform fluence from any given position of the treatment beam to the patient [3]. The dose distribution needs to be calculated with a radiation treatment planning system (RTPS), and patient-specific quality assurance (QA) is needed to validate the dose distribution. Dose distributions can be measured by either using a chamber (i.e., point dosimetry) or a film or diode array detector (i.e., two-dimensional dosimetry) [4,5]. For patient-specific QA using a film or array detector, the gamma index is currently used extensively to validate the difference between the calculated and measured doses [6,7].

In recent years, various cases of radiotherapy using 3D printing technology have been reported. For instance, Ehler et al. [8] constructed the head and neck components of the Rando phantom (The Phantom Laboratory, Salem NY) with a 3D printer. Park et al. [9] created a 3D customized bolus and used it as a compensator in IMRT of a patient with Kimura’s disease involving the auricle. Choi et al. [10] utilized a 3D printer to evaluate the effectiveness and accuracy of a patient-specific bolus in electron beam therapy, while Kim et al. [11] created a customized bolus with a 3D printer to analyze its effectiveness in comparison with the conventional Superflab bolus in photon beam radiotherapy. For proton therapy, Ju et al. [12] concluded that the physical accuracy and dosimetric characteristics of a proton range compensator (RC) created with a 3D printer and the conventional RC constructed with a computerized milling machine are comparable. Our institution [13] recently fabricated a customized 3D bolus for an irregular surface by using a 3D scanner and 3D printer.

Ehler et al. [8] pointed out that a QA method based on using a 3D phantom constructed with a 3D printer provides a better end-to-end test than traditional per-patient quality control tests because it matches the clinical patient workflow.

To realize the advantages of this patient-specific phantom using a 3D printer, this study evaluated the feasibility of using a 3D-printed anthropomorphic patient-specific head phantom of a head and neck cancer patient for patient-specific QA in IMRT.

## Materials and methods

### Construction procedure for a patient-specific head phantom

**Fig 1:**
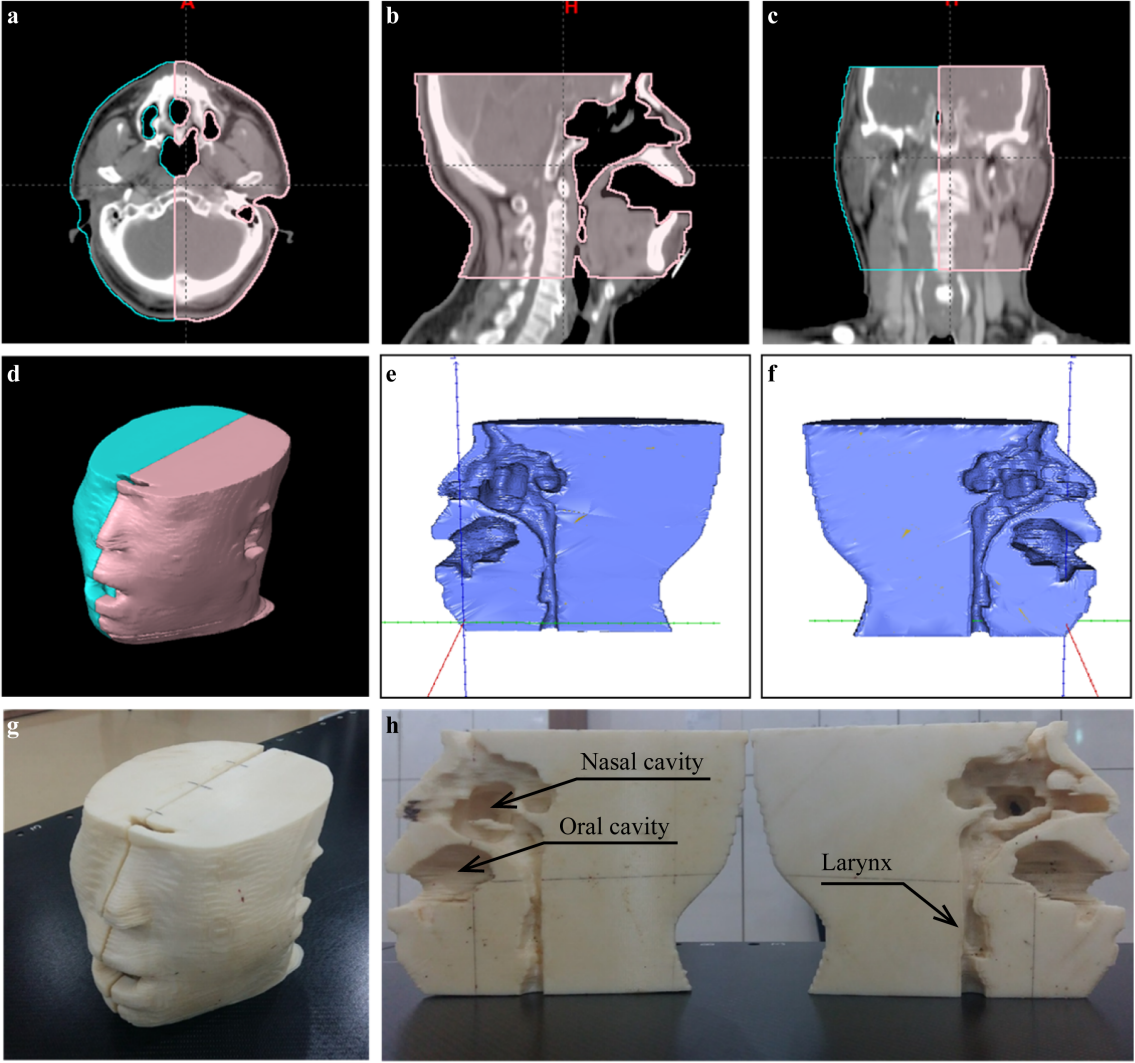
Images for a head phantom. (a) Transversal, (b) sagittal, and (c) frontal plane images contoured by auto segmentation for the left and right head phantoms on the Eclipse RTPS from the DICOM CT images. (d) Model view reconstructed by the RTPS for the left and right head phantoms. (e) 3D rendering of the left head phantom. (f) 3D rendering of the right head phantom displayed by STL Viewer. (g) Image of the 3D-printed anthropomorphic patient-specific head phantom. (h) Patient-specific head phantom including air cavities such as the nasal cavity, oral cavity, and larynx.

The computed tomography (CT) image used in this study was a Digital Imaging and Communications in Medicine (DICOM) file intended for use in the radiotherapy treatment plan for head and neck cancer patients. The CT image was scanned at 3 mm intervals with a Philips Big Bore Brilliance CT Scanner (Philips Medical, Eindhoven, Netherlands). The scanned CT image was then sent to an Eclipse 8.6 RTPS (Varian Medical System Inc., Palo Alto, CA). The image was scanned in order to increase the smoothing level of the 3D-printed head phantom and reconstructed in intervals of 0.5 mm at the Eclipse RTPS because of its thickness. The “search body” function in the contouring tab of the Eclipse system was used to contour the body automatically. Just like that used clinically for patient CT, the lower threshold was set to −300 Hounsfield units (HU). The contoured body structure was partitioned to the left and right to measure the radiation dose, as shown in Fig 1(A)–1(D). Because the STereoLithography (STL) file format was required for 3D printing, the left and right head phantoms contoured from the RTPS were converted from DICOM to STL format by using the SlicerRT toolkit in the open-source 3D Slicer (version 4.4; www.slicer.org) Fig 1(E) and 1(F) display the converted file with STL Viewer.

An anthropomorphic patient-specific head phantom was constructed by using the fused deposition modeling (FDM) method with a Dimension 1200 Series SST printer (Stratasys, Eden Prairie, MN). ABSplus (Stratasys, Eden Prairie, MN) was chosen as the material to construct the head phantom. Fig 1(G) and 1(H) show the constructed anthropomorphic patient-specific head phantom. Structures such as the oral cavity, nasal cavity, and larynx were left as cavities.

### CT scan using a patient-specific head phantom and treatment plan

**Fig 2:**
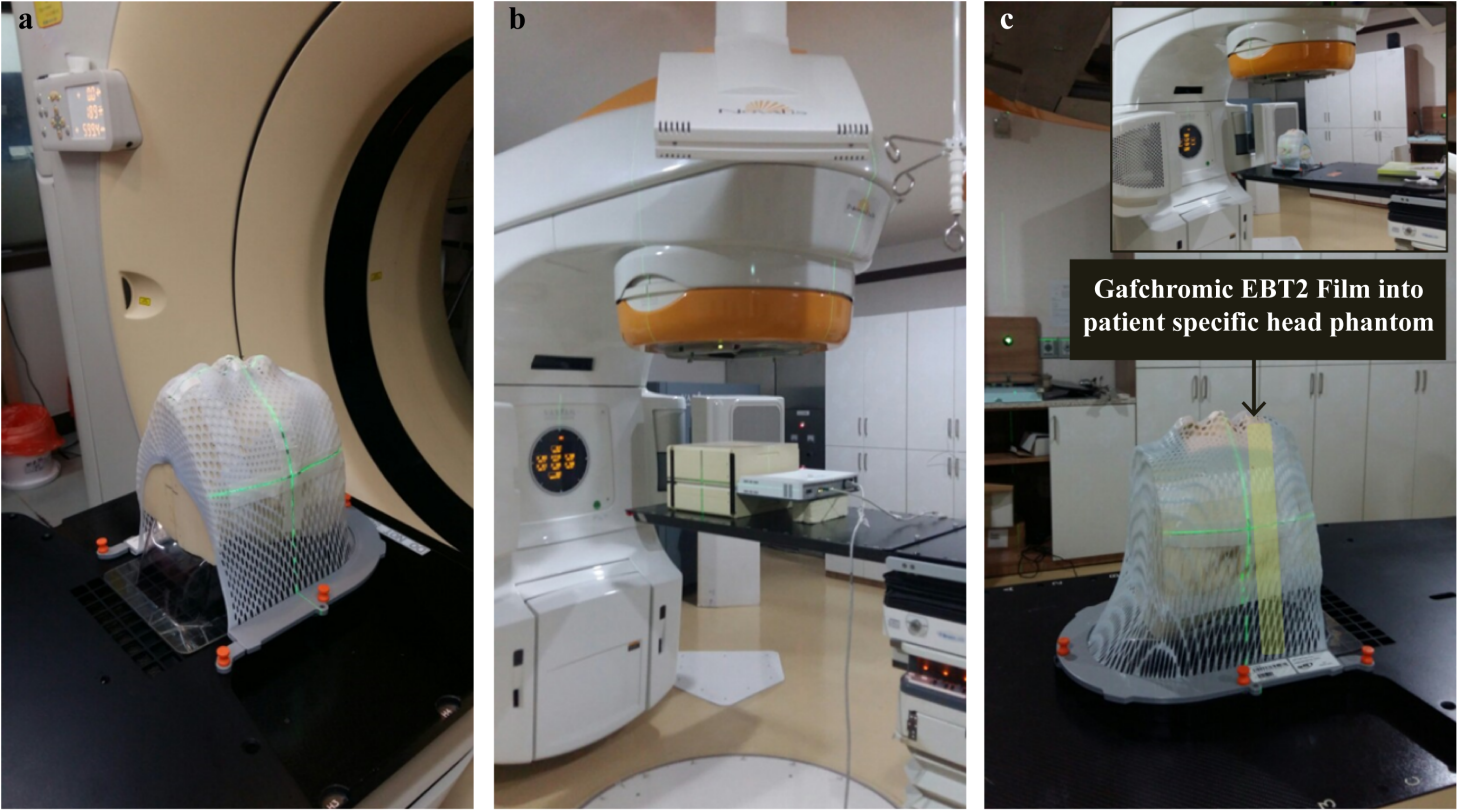
Scanning of the patient-specific head phantom. (a) Patient-specific head phantom immobilized with a DUON^TM^ mask to fix the phantom scanned with the CT scanner, (b) QA verification with I’mRT MatriXX into MULTICube, and (c) QA verification with the patient-specific head phantom and Gafchromic EBT2 film.

**Fig 3:**
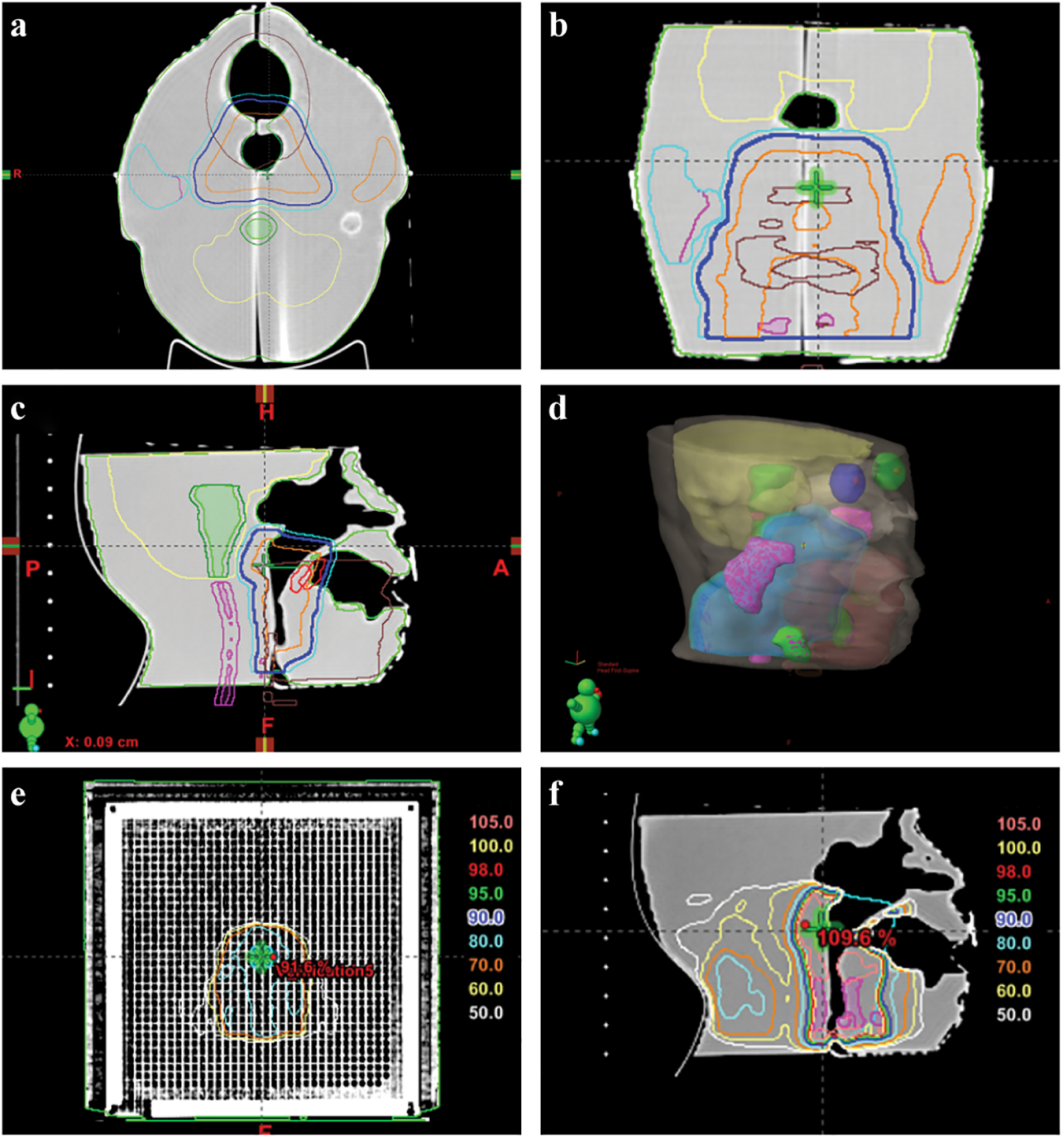
Treatment plan for the patient-specific head phantom. (a) Transversal (b) frontal, and (c) sagittal plane images and (d) model view image of the CT scanned with the patient-specific head phantom and copied to the RT structure by fusing information from the real patient and patient-specific head phantom. Dose distributions calculated with (e) I’mRT MatriXX in the frontal plane and (f) the 3D-printed patient-specific head phantom in the sagittal plane.

In order to obtain the CT DICOM for the radiotherapy treatment plan using the patient-specific head phantom in Fig 2(A), the image was scanned at a thickness of 1 mm. For other conditions, the same protocol was used for the head phantom as that for a CT of a patient’s head. The patient-specific head phantom was fixed into position with a DUON^TM^ mask (Orfit Industries, Wijnegem, Belgium) (Fig 2(B) and 2(C)). The CT images of a real patient (Fig 1(A)–1(C)) and the patient-specific head phantom (Fig 3(A)–3(C)) were fused, and the “copy all structure” function was used to override the head phantom with the structure contoured from the patient. Fig 3(D) shows the model view of the fused CT image.

The treatment plan for the patient-specific head phantom was formulated by performing fixed-gantry IMRT at the angles 0°, 40°, 80°, 120°, 160°, 200°, 240°, 280°, and 320°. In this treatment plan, the same tolerance and priority values used for a real patient’s target and normal organs were applied and re-optimized. Fig 3(F) shows the dose distribution calculated for the 3D-printed patient-specific head phantom in the sagittal plane. The treatment plan re-optimized for the head phantom was also transferred to I’mRT MatriXX (IBA Dosimetry GmbH) for calculation. Fig 3(E) shows the dose distributions calculated with I’mRT MatriXX in the frontal plane. The ion chambers were arranged in a 2D array on the frontal plane, which made it possible to compare the measured and calculated doses. The sagittal plane of the patient-specific head phantom was constructed so that a Gafchromic EBT2 film could be inserted for comparison of the measured and calculated dose distributions.

### Evaluation of the dose distributions with I’mRT MatriXX and a patient-specific head phantom: gamma index (γ)

To verify the calculated and measured dose distributions, we used I’mRT MatriXX with the Plastic Water^®^ phantom MULTICube (IBA Dosimetry GmbH) and the 3D-printed patient specific head phantom. In recent years, the gamma index has been widely used to compare calculated and measured doses [6,7,14].

Janusz et al. [14] defined the gamma index (γ) to compare the distributions of the calculated and measured doses as follows:

$$\gamma (r_{m},r_{c})=\sqrt{\frac{r^{2}\;(r_{m},r_{c})}{\Delta {d^{2}}_{M}}+\frac{\delta^{2}\;(r_{m},r_{c})}{\Delta {D^{2}}_{M}}}$$ (1)

where

$$r(r_{m},r)=\lfloor r-r_{m}\rfloor$$ (2)

and

$$\delta (r_{m},r)=D(r)-D_{m}\;(r_{m})$$ (3)

is the dose difference (DD) at the position r_*m*_. Here, r_*m*_ is a single measurement point, *r*_*c*_ is the spatial location of the calculated distribution relative to the measured point, Δ*d*_*M*_ is the passing criterion for the distance-to-agreement (DTA), and Δ*D*_*M*_ is the passing criterion for the DD. The calculation passes if γ ≤ 1 and fails if γ > 1. In order to compare the calculated and measured doses for gamma evaluation, the acceptable DD and DTA values were set to 3% and 3 mm, respectively.

### Patient-specific quality assurance with I’mRT MatriXX and a patient-specific head phantom

#### I’mRT Matrixx

A Novalis Tx linear accelerator system (Varian Medical Systems, CA, USA and BrainLAB, Feldkirchen, Germany) with an HD-120 high-definition multi-leaf collimator (HD-MLC) was used for verifying the measured and calculated dose distributions for IMRT. The Eclipse 8.6 RTPS and anisotropic analytical algorithm (AAA) were used to calculate the radiation dose with a grid size of 2.5 mm and inhomogeneity correction. Our previous study [15] and Park et al. [16] showed that the AAA algorithm with a grid size of 2.5 mm is sufficiently accurate for low-density regions such as the lungs. Measurements were made by using I’mRT MatriXX with MULTICube, and the source-to-axis distance (SAD) was set to 100 cm. The calculated and measured radiation doses with I’mRT MatriXX were carried out at a 0° gantry angle as stipulated by the internal QA protocol.

#### Patient-specific head phantom and Gafchromic EBT2 film

The radiation dose was calculated in the same manner as that used with I’mRT MatriXX but on the sagittal plane because the radiation dose was to be measured in this plane. The calibration process for the Gafchromic EBT2 film was done in a previous study [17]. Among three channels (red, green, and blue), the red calibration curve was selected to compare the calculated and measured radiation doses for the patient-specific head phantom because the analog-to-digital (ADC) converter value for the red channel was least sensitive to changes in the radiation dosage. The calculation and measurement of the radiation dose with the 3D-printed patient-specific head phantom and Gafchromic EBT2 film were performed by using the fixed-gantry IMRT technique with 6 MV photons at gantry angles of 0°, 40°, 80°, 120°, 160°, 200°, 240°, 280°, and 320°.

## Results

### 3D-printed patient-specific head phantom

**Fig 4:**
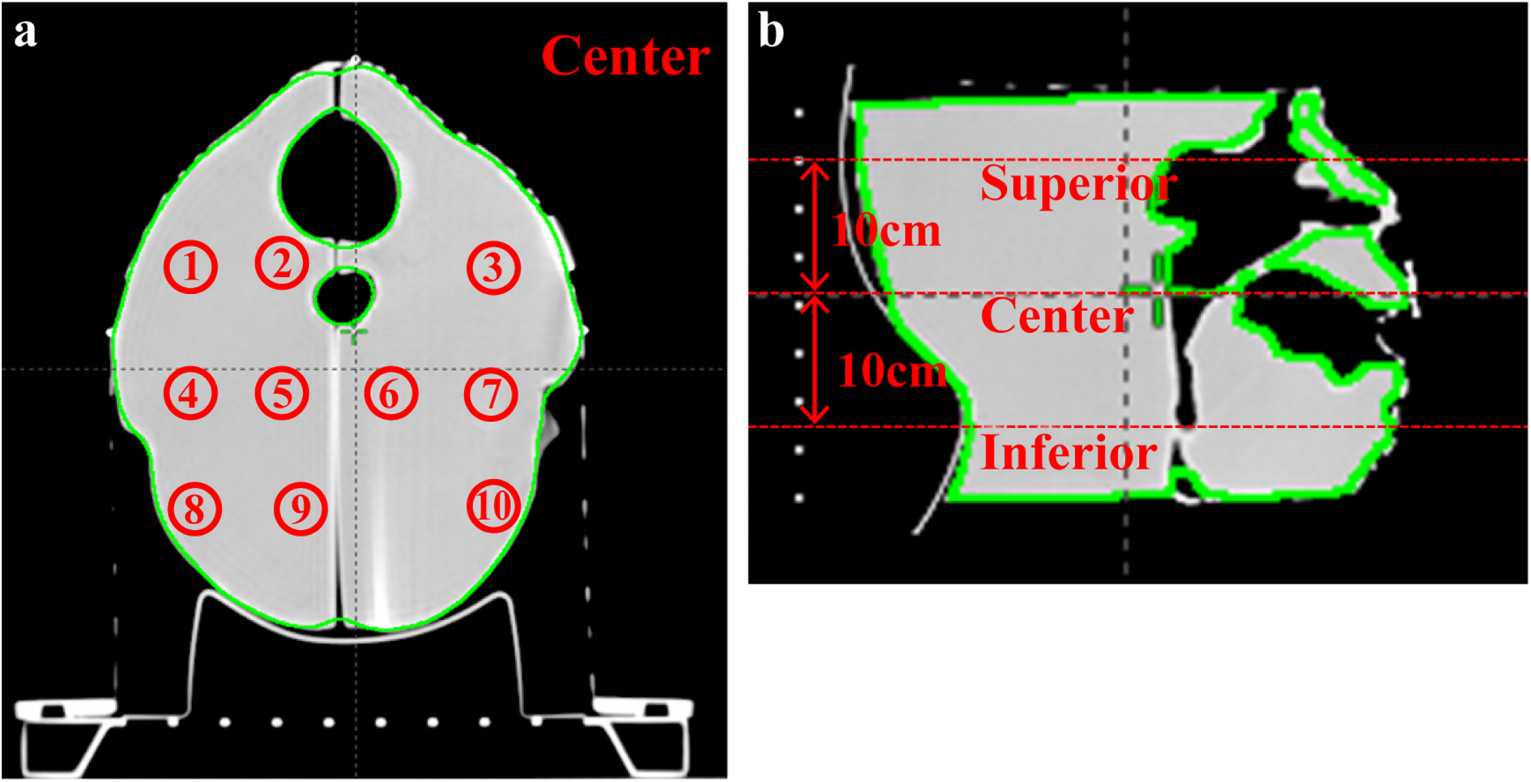
Superior (+10 cm), center (0 cm), and inferior (−10 cm) measurement positions for the HU of the patient-specific head phantom. (a) Transversal and (b) sagittal plane images.

Fig 4 shows the CT image of the 3D-printed patient-specific head phantom scanned at 1-mm intervals on the (a) transversal and (b) sagittal planes. In order to investigate the homogeneity of the HU of the head phantom, a point marked during the CT scan was designated as the center, as shown in Fig 4(B). Ten points were selected at the center, superior (+10 cm), and inferior (−10 cm) positions for HU measurement. S1 Table presents the results. The mean HU at the center was −340 with a standard deviation of 11.6, while the mean and standard deviation at the superior position were −338 and 14.6, respectively. The mean and standard deviation at the inferior position were −339 and 9.9, respectively. Overall, the HU for the patient-specific head phantom was largely homogeneous.

### Test for dose accuracy in I’mRT MatriXX

**Fig 5:**
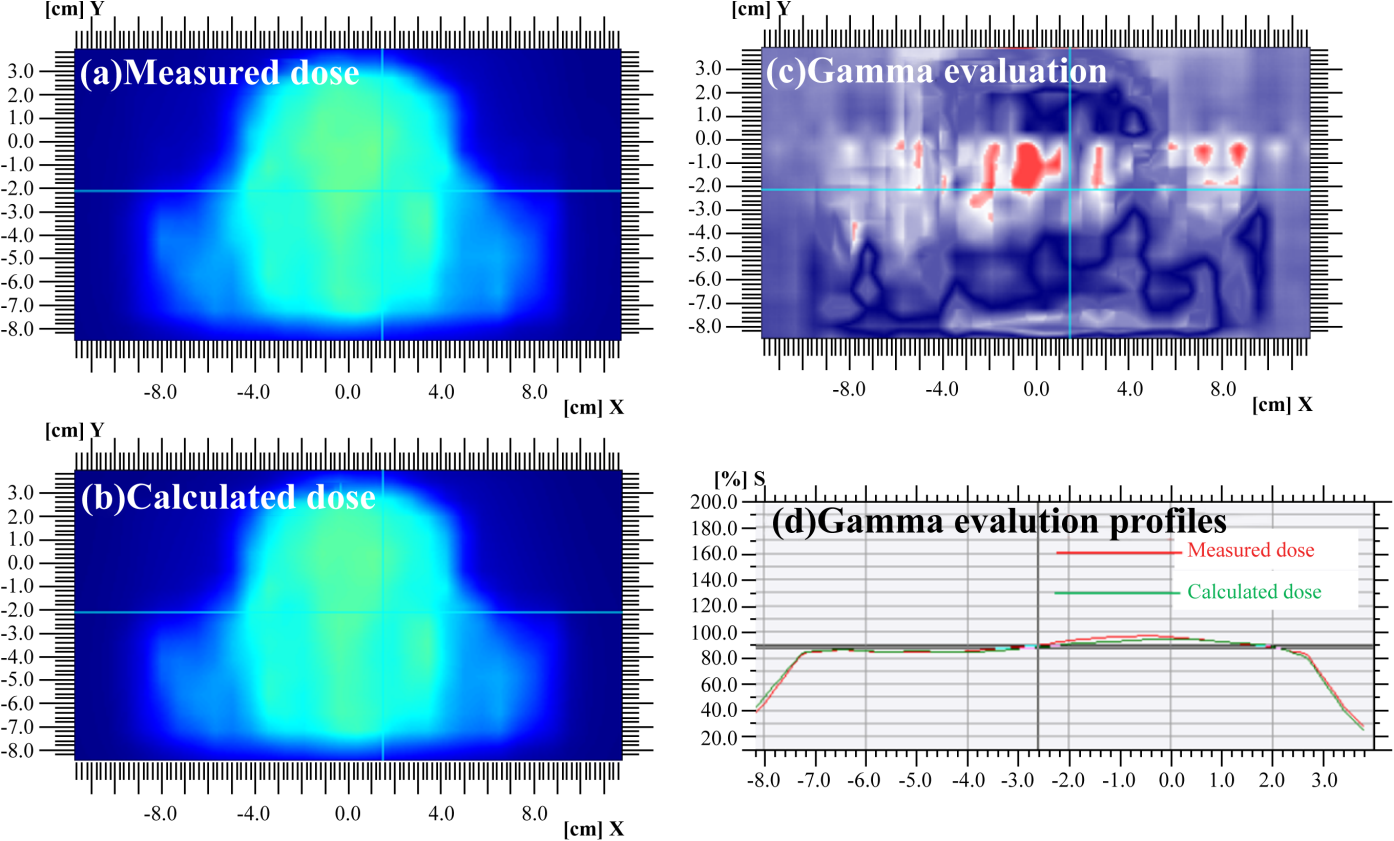
Dose profiles with I’mRT MatriXX. (a) Measured dose, (b) calculated dose, (c) gamma maps (3%/3 mm), and (d) gamma evaluation profiles using I’mRT MatriXX in the frontal plane. The red and green lines show the measured and calculated dose profiles, respectively.

Fig 5 compares the calculated and measured doses with I’mRT MatriXX by using the gamma index (γ). Fig 5(A) shows the calculated dose distribution at the frontal plane, while Fig 5(B) shows the measured dose distribution. Fig 5(C) is a gamma map in which the red regions indicate where γ ≥ 1, and the bluish-violet regions indicate where γ < 1. The acceptance DD and DTA used for gamma evaluation were set to 3% and 3 mm, respectively [4]. Fig 5(D) represents the gamma evaluation profiles. The red line represents the distribution of the measured dose, while the green line indicates the distribution of the calculated dose. The gamma passing rate (i.e., γ ≤ 1), was 97.28%, and the gamma failure rate (i.e., γ > 1) was 2.72%.

### Test for dose accuracy with the 3D-printed patient-specific head phantom

**Fig 6:**
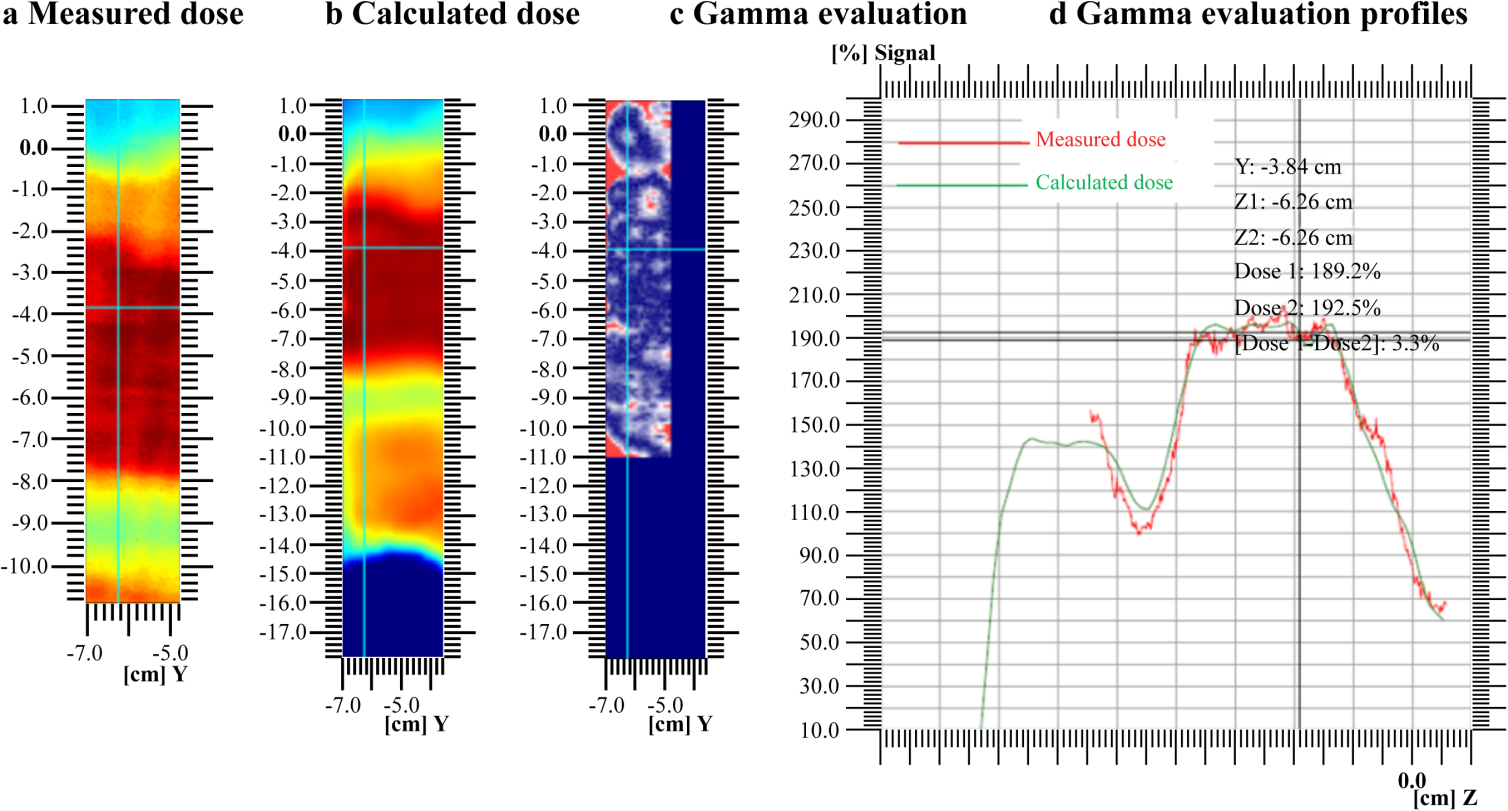
Dose profiles with the 3D-printed patient-specific head phantom and Gafchromic EBT2 film. (a) Measured dose, (b) calculated dose, (c) gamma maps (3%/3 mm), and (d) gamma evaluation profiles using the 3D-printed anthropomorphic patient-specific head phantom and Gafchromic EBT2 film in the sagittal plane. The red and green lines show the measured and calculated dose profiles, respectively.

Fig 6 compares the calculated and measured doses with the 3D-printed patient-specific head phantom and Gafchromic EBT2 film using the γ index. Fig 6(A) shows the calculated dose distribution at the sagittal plane, and Fig 6(B) illustrates the measured dose distribution. Fig 6(C) is a gamma map in which the regions in red indicate where γ ≥ 1, while the bluish-violet regions indicate where γ < 1 and Fig 6(D) shows the gamma evaluation profiles using the 3D-printed anthropomorphic patient-specific head phantom and Gafchromic EBT2 film in the sagittal plane. Similar to I’mRT MatriXX, the same values for the acceptance DD (3%) and DTA (3 mm) were applied in calculations with the patient-specific head phantom. The results produced a gamma passing rate (γ ≤ 1) of 95.97% and failure rate (γ > 1) of 4.03%.

## Discussion

In this study, the feasibility of using a 3D-printed patient-specific head phantom to compare the calculated and measured radiation doses was verified. We fabricated an anthropomorphic patient-specific head phantom by using 3D printing technology to verify the calculated and measured radiation doses. We compared the conventional QA measured with a 2D diode array and the head phantom fabricated with a 3D printer.

While the radiation doses in radiotherapy treatments are often measured by using an ion chamber, diode 2D array, and films [4,5,7,8], there is little in the literature on utilizing a patient-specific phantom to calculate and measure radiation doses. In this research, a patient-specific head phantom was constructed from a DICOM file by using 3D printing technology, which has seen rapid advances in recent years.

In summary, the performance with the patient-specific head phantom was compared to that of I’mRT MatriXX, which is a well-established IMRT QA technique. The 2D array and patient-specific head phantom were analyzed individually by using the gamma index. At the acceptance DD and DTA values of 3% and 3 mm, respectively, the gamma passing rate with γ ≤ 1 was 97.28% with I’mRT MatriXX and 95.97% with the patient-specific head phantom for a difference of approximately 1.31%. Consequently, our results showed that the 3D-printed patient-specific head phantom has great potential for patient-specific QA of intensity-modulated radiotherapy.

In a similar study, Ehler et al. [8] created an anthropomorphic Rando phantom with heterogeneous tissue equivalence in order to compare the measured and calculated radiation doses, where FDM was applied to acrylonitrile butadiene styrene (ABS). They intended to realize dosimetric measurements that would not be possible in vivo and to avoid patient privacy issues during research. A nine-field IMRT plan was employed with a traditional verification method using the planar and cylindrical phantoms MapCHECK2 and ArcCHECK (Sun Nuclear Corp, Melbourne, FL). The mean percentage difference between the calculated and measured doses for the Rando phantom was 1.9%±2.8%, whereas the value with the 3D printed phantom was −0.1%±4.9%, so the difference between the two types of phantoms was minimal. Thus, they concluded that patient-specific phantoms fabricated by a 3D printer can be used for dosimetric verifications between the measured and calculated doses.

Joseph et al. [18] recently reported that polystyrene can be used to fabricate a 3D low-density phantom for radiotherapy QA with a relative electron density of 0.18–0.75 by using the ORION Delta 3D (SeeMeCNC Indiana) phantom. They concluded that 3D printing allows the fabrication of variable-density phantoms for radiotherapy QA in low-density regions from the correlation curve between the 3D printer infill value and relative electron density. However, they noted limitations for use in phantoms with an exceedingly low density.

These variable-density issues have been a great challenge in applying 3D printing technology to patient-specific QA of radiotherapy because most commercially available 3D printers currently support only one material at the time of fabrication. Thus, one limitation of our study was that we used only a single material (ABSplus) for the fabrication of the head phantom, while the body of a real patient consists of various types of organs that produce a much wider range of HU values.

In future work, we plan to fabricate a variable-density patient-specific phantom to compare the calculated and measured doses. We also want to investigate the linear attenuation coefficient for radiotherapy by varying the different materials used in 3D printing.

Nevertheless, the 3D-printed phantom in this study can provide a better end-to-end test than the traditional quality control test.

## Conclusions

In this research, a patient-specific head phantom identical to the head of a real patient was constructed with a 3D printer by using the patient’s CT image. The phantom can be utilized for dosimetric verification that must be performed prior to complex radiotherapy treatment, such as IMRT and VMAT. In future work, we will research fabricating phantoms with variable density to simulate various components of the human body, including the lungs, fat, blood, muscle, soft tissue, and bones when comparing the calculated and measured radiation doses.

## Supporting information

S1 Table Measured Hounsfield units (HU) in the superior, center, and inferior positions with the patient-specific head phantom. (DOCX)

## References

[1] Oh SA, Kang MK, Kim SK, Yea JW. Comparison of IMRT and VMAT techniques in spine stereotactic radiosurgery with international spine radiosurgery consortium consensus guidelines. Prog Med Phys 2013; 24: 145–153.

[2] Oh SA, Kang MK, Yea JW, Kim SK, Oh YK. Study of the penumbra for high-energy photon beams with Gafchromic™ EBT2 films. J Korean Phys Soc 2012; 60: 1973–1976.

[3] Khan FM, Gibbons JP. Khan’s the physics of radiation therapy 5th ed. Philadelphia: Lippincott Williams & Wilkins; 2014.

[4] Ezzell GA, Burmeister JW, Dogan N, LoSasso TJ, Mechalakos JG, Mihailidis D, et al IMRT commissioning: multiple institution planning and dosimetry comparisons, a report from AAPM Task Group 119. Med Phys 2009; 36: 5359–5373. doi: 10.1118/1.3238104 19994544 10.1118/1.3238104

[5] Low DA, Moran JM, Dempsey JF, Dong L, Oldham M. Dosimetry tools and techniques for IMRT. Med Phys 2011; 38: 1313–1338. doi: 10.1118/1.3514120 21520843 10.1118/1.3514120

[6] Low DA, Harms WB, Mutic S, Purdy JA. A technique for the quantitative evaluation of dose distributions. Med Phys 1998; 25: 656–661. doi: 10.1118/1.598248 9608475 10.1118/1.598248

[7] Oh SA, Kim SK, Kang MK, Yea JW, Kim EC. Dosimetric verification of enhanced dynamic wedges by a 2D ion chamber array. J Korean Phys Soc 2013; 63: 2215–2219.

[8] Ehler ED, Barney BM, Higgins PD, Dusenbery KE. Patient specific 3D printed phantom for IMRT quality assurance. Phys Med Biol 2014; 59: 5763 doi: 10.1088/0031-9155/59/19/5763 25207965 10.1088/0031-9155/59/19/5763

[9] Park J, Yea J. Three-dimensional customized bolus for intensity-modulated radiotherapy in a patient with Kimura's disease involving the auricle. Cancer/Radiothérapie 2016; 20: 205–209. 10.1016/j.canrad.2015.11.003 27020714

[10] Choi WK, Chun JC, Ju SG, Min BJ, Park SY, Nam HR, et al Efficacy and accuracy of patient specific customize bolus using a 3-dimensional printer for electron beam therapy. Prog Med Phys 2016; 27: 64–71.

[11] Kim S-W, Shin H-J, Kay CS, Son SH. A customized bolus produced using a 3-dimensional printer for radiotherapy. PloS One 2014; 9: e110746 doi: 10.1371/journal.pone.0110746 25337700 10.1371/journal.pone.0110746 PMC4206462

[12] Ju SG, Kim MK, Hong C-S, Kim JS, Han Y, Choi DH, et al New technique for developing a proton range compensator with use of a 3-dimensional printer. Int J Radiat Oncol* Biol* Phys 2014; 88: 453–458. 10.1016/j.ijrobp.2013.10.024 24315564

[13] Park JW, Oh SA, Yea JW, Kang MK. Fabrication of malleable three-dimensional-printed customized bolus using three-dimensional scanner. PloS One 2017; 12: e0177562 doi: 10.1371/journal.pone.0177562 28494012 10.1371/journal.pone.0177562 PMC5426771

[14] Winiecki J, Morgaś T, Majewska K, Drzewiecka B. The gamma evaluation method as a routine QA procedure of IMRT. Rep Pract Oncol Radiother 2009; 14: 162–168.

[15] Oh SA, Kang MK, Yea JW, Kim SH, Kim KH, Kim SK. Comparison of intensity modulated radiation therapy dose calculations with a PBC and AAA algorithms in the lung cancer. Korean J Med Phys 2012; 23: 48–53.

[16] Park S-Y, Park JM, Choi CH, Chun M, Kim J-i. Dosimetric validation of the Acuros XB advanced dose calculation algorithm for volumetric modulated arc therapy plans. Prog Med Phys 2016; 27: 180–188.

[17] Oh SA, Yea JW, Lee R, Park HB, Kim SK. Dosimetric verifications of the output factors in the small field less than 3 cm^2^ using the gafchromic EBT2 films and the various detectors. Prog Med Phys 2014; 25: 218–224.

[18] Madamesila J, McGeachy P, Barajas JEV, Khan R. Characterizing 3D printing in the fabrication of variable density phantoms for quality assurance of radiotherapy. Phys Med 2016; 32: 242–247. doi: 10.1016/j.ejmp.2015.09.013 26508016 10.1016/j.ejmp.2015.09.013

