# Supplementary material for: Feasibility of a 3D-printed anthropomorphic patient-specific head phantom for patient-specific quality assurance of intensity-modulated radiotherapy
Source: PLoS One. 2017 Jul 20;12(7):e0181560. doi: 10.1371/journal.pone.0181560 (PMC5519219; doi:10.1371/journal.pone.0181560)
Supplement: S1 Table — (DOCX) [file pone.0181560.s001.docx]

**S1 Table**

Measured Hounsfield units (HU) in the superior, center, and inferior positions with the patient−specific head phantom.

| No. | Center | Superior (+10 cm) | Inferior (−10 cm) |
| --- | --- | --- | --- |
| 1 | −356 | −352 | −333 |
| 2 | −354 | −336 | −338 |
| 3 | −346 | −346 | −353 |
| 4 | −319 | −325 | −348 |
| 5 | −342 | −351 | −338 |
| 6 | −349 | −333 | −352 |
| 7 | −336 | −332 | −328 |
| 8 | −328 | −360 | −345 |
| 9 | −334 | −338 | −331 |
| 10 | −339 | −310 | −326 |
| Average | −340 | −338 | −339 |
| Standard deviation | 11.6 | 14.6 | 9.9 |
